# CIRCUS: CIRCUlating tumour cells in soft tissue Sarcoma - a short report

**DOI:** 10.20517/cdr.2024.149

**Published:** 2024-12-13

**Authors:** Robin J. Young, Joanna E. Chowdry, Denis Cochonneau, Dominique Heymann

**Affiliations:** ^1^Weston Park Cancer Centre, Sheffield Teaching Hospitals NHS Foundation Trust, Sheffield S10 2SJ, UK.; ^2^School of Medicine and Population Health, University of Sheffield, Sheffield S10 2RX, UK.; ^3^Tumour Heterogeneity and Precision Medicine Laboratory, Institut de Cancérologie de l’ouest, Saint-Herblain 44805, France.; ^4^Unité Mixte de Recherche n°6286, Centre National de la Recherche Scientifique, Nantes Université, Nantes 44322, France.

**Keywords:** Circulating tumour cells, soft tissue sarcoma, metastasis

## Abstract

**Aims:** Circulating tumour cells (CTCs) can be detected in peripheral blood using their physical properties (increased size and less deformable than normal circulating blood cells) or using cell surface markers. The study of these CTCs should provide important insights into tumour biology, including mechanisms of drug resistance. We performed a pilot study (IRAS ID: 235459) to evaluate if CTCs could be isolated from peripheral blood samples collected from soft tissue sarcoma (STS) patients.

**Methods:** We used a combined approach that first enriched samples for CTCs using a microfluidic cassette via Parosrtix^TM^PR1, and then sorted cells stained for vimentin and cytokeratin using the DEPArray^TM^. The total circulating cell-free DNA (cfDNA) level was also analysed. Data were correlated with clinical parameters.

**Results:** 13 patients were recruited to this study: 7 patients with localised disease and 6 patients with metastatic disease. CTCs exhibited a high heterogeneity based on their expression of mesenchymal and epithelial markers. There was no significant difference in the number of CTCs between patients with localised versus metastatic disease. We observed no correlation between CTC numbers and cfDNA; however, the number of CTCs did correlate with primary tumour size.

**Conclusion:** The present study demonstrates the presence of CTCs in STS patients with localised and advanced disease. Further and larger studies are needed to characterise STS CTCs and to evaluate their prognostic significance.

## INTRODUCTION

Sarcomas are a rare, heterogeneous group of tumours of mesenchymal origin^[1]^. The biology of sarcoma subtypes varies widely; however, all sarcomas preferentially metastasise to the lungs, typically via haematological spread. The risk of metastases is dependent on various tumour-specific factors, including tumour histology, size, and grade. Approximately 50% of patients with localised high-risk extremity soft tissue sarcoma (STS) eventually develop metastatic disease^[2]^. Once metastases occur, treatment is usually palliative and the prognosis is poor. A deeper understanding of the metastatic process is needed to develop more effective treatments for STS.

Cancer cells that escape the primary tumour and intravasate into the bloodstream become circulating tumour cells (CTCs) that have the potential to seed into distant metastases^[3]^. CTCs can be detected in the peripheral blood, and the number of CTCs may correlate with clinical endpoints such as the burden of metastatic disease and response to treatment^[4]^. As a window into the metastatic niche, the study of CTCs should improve our understanding of the metastatic process, and represent a potential tool for studying mechanisms of drug resistance^[5]^. To date, most studies on CTCs have focused on patients with carcinomas, utilising tumour cell expression of cytokeratins such as epithelial cell adhesion molecule (EpCAM) to capture CTCs. In contrast, research on CTCs in sarcomas remains limited. This is partly due to the rarity and heterogeneity of sarcomas, as well as the absence of a sarcoma-specific CTC marker. However, vimentin (Vim) has recently been suggested as one such marker^[6]^. Additionally, because CTCs are often larger and less deformable than normal circulating blood cells, cell size has been proposed as another distinguishing feature for their separation^[7]^.

We developed a workflow to isolate and analyse sarcoma CTCs based on tumour size and performed a pilot study (IRAS ID: 235459), which we report here, to isolate, quantify, and characterise CTCs in peripheral blood samples collected from patients presenting to the Sheffield Sarcoma Service with either localised or advanced extremity STS.

## METHODS

This research study was approved by the London - Surrey Borders Research Ethics Committee and Health Research Authority (IRAS project ID: 235459, REC reference 18/LO/1812) and sponsored by Sheffield Teaching Hospitals NHS Foundation Trust (STH: 19860).

Patients presenting to the Sheffield STS Service with a new diagnosis of localised or advanced extremity STS were eligible. Patient management was not protocol-defined; treatment and follow-up schedules were based on local and national guidelines^[3]^. Patient demographics, tumour histology, staging, and disease-free survival were extracted from medical records. Written informed consent for the study and CTC blood sample collections were obtained before initiation of any treatment (surgery, radiotherapy or chemotherapy). Twenty mL peripheral blood samples were collected from patients into 2 mL × 10 mL Streck^TM^ (Streck, USA) tubes and gently inverted ten times after collection to ensure proper mixing with the preservative. The samples were enriched for CTCs within 96 h of collection using a Parsortix^TM^PR1 instrument (Angle Ltd, UK); the blood sample was loaded onto a microfiltration cassette, which traps CTCs owing to their larger size in comparison to other blood components. The CTC isolate was then fixed in formaldehyde and stored at 4 ^o^C until analysis. CTCs were then separated from other cells in the isolate and quantified using dielectrophoresis with the DEPArray^TM^ (Menarini, Italy). Commercially available antibodies were employed to exclude contaminating white blood cell populations, using the white blood cell marker CD45 (1/20, PerCP/Cy5.5-mouse IgG1k anti-human, clone HI30, Biolegend, USA). CTC expression of Vim was evaluated using anti-Vim antibody (10 μg/mL, mouse anti-IgG2a anti-human, clone VIM3B4, Dako, France) associated with an AF647-goat anti-mouse IgG2a (1/1,000, Invitrogen, Fisher Scientific, France), and pan-cytokeratin (CK) expression evaluated using an anti-AE1/AE3 antibody (5 μg/mL, AF488-mouse IgG1k anti-human, clone C-11, Biolegend).

Plasma extracted at the enrichment phase was aliquoted and stored at -20 ^o^C. Total cell-free (cfDNA) was isolated by NucleoSnap^TM^ cfDNA columns (Macherey-Nagel, France) and quantified by NanoDrop^TM^ (Ozyme, France) according to the manufacturer’s instructions; cfDNA levels averaged across paired replicate samples were reported.

### Statistical analysis

Non-parametric tests were performed; the Mann-Whitney *U* test was used to compare populations and Spearman’s Rho for correlations. *P* < 0.05 was considered significant.

## RESULTS

Thirteen patients [7 male, 6 female, median age 58 years (39-82)] were recruited from 2019-2022: 7 patients with localised disease and 6 patients with advanced disease. Tumour histology included 6 cases of undifferentiated pleomorphic sarcoma (UPS), 2 of myxofibrosarcoma, 2 of myxoid liposarcoma, 1 of de-differentiated liposarcoma, 1 of leiomyosarcoma, and 1 of angiosarcoma. All 6 patients with metastatic disease had UPS [Tables 1 and 2].

**Table 1:** Clinical and biological parameters of the seven patients recruited with localised disease

**Age (yrs)**	**Sex**	**Tumour histology**	**Primary tumour site**	**Maximum tumour size (mm)**	**Trojani grade**	**Peri-operative RT given**	**Peri-operative CT given**	**Disease-free at 3 years**	**Number of CTCs**				**cfDNA (ng/μL)**
									**Vim^+^ CK^+^**	**Vim^-^ CK^+^**	**Vim^+^ CK^-^**	**Total**	
71	M	MFS	Rt arm	55	2	Yes	No	Yes	1	9	2	12	0.12
54	M	MLPS	Rt thigh	170	1	Yes	No	Yes	12	5	5	22	0.11
39	M	MLPS	Rt lower leg	118	2	Yes	No	No	1	0	2	3	0.19
58	F	AS	Rt breast	190	3	No	No	No	67	29	14	110	0.12
42	F	MFS	Lt knee	50	2	Yes	No	No	0	1	0	1	0.07
69	F	LMS	Lt thigh	29	3	No	No	Yes	1	1	0	2	0.32
82	F	DDLPS	Lt thigh	40	2	Yes	No	No	1	0	0	1	2.00

CTCs: Circulating tumour cells; Vim: vimentin; CK: cytokeratin; cfDNA: cell-free DNA; AS: angiosarcoma; CT: chemotherapy; DDLPS: de-differentiated liposarcoma; LMS: leiomyosarcoma; MFS: myxofibrosarcoma; MLPS: myxoid liposarcoma; RT: radiotherapy; Rt: right; Lt: left; M: male; F: female.

**Table 2:** Clinical and biological parameters of the six patients recruited with advanced disease

**Age (yrs)**	**Sex**	**Tumour histology**	**TNM stage**	**Sites of disease**	**Primary tumour size (mm)**	**Trojani grade**	**Number of CTCs**				**cfDNA (ng/μL)**
							**Vim^+^ CK^+^**	**Vim^-^ CK^+^**	**Vim^+^ CK^-^**	**Total**	
73	M	UPS	TX N0 M1	Multiple lung + bilateral adrenal metastases	-	3	13	22	0	35	0.62
58	M	UPS	T4 N1 M0	Primary + Rt iliac LNs	220	2	42	91	0	133	NA
57	M	UPS	T4 N1 M1	Primary + Rt iliac LNs + solitary lung metastasis	170	2	0	2	10	12	0.23
62	F	UPS	T1 N0 M1	Local recurrence + multiple lung metastases	43	3	5	9	3	17	0.10
61	M	UPS	TX N0 M1	Soft tissue (regional recurrence)	-	3	2	0	0	2	0.32
42	F	UPS	TX N0 M1	Multiple lung metastases	-	3	3	0	0	3	NA

CTCs: Circulating tumour cells; Vim: vimentin; CK: cytokeratin; cfDNA: cell-free DNA; UPS: undifferentiated pleomorphic sarcoma; LNs: lymph nodes; Rt: right; NA: sample not available; M: male; F: female.

**Figure 1:**
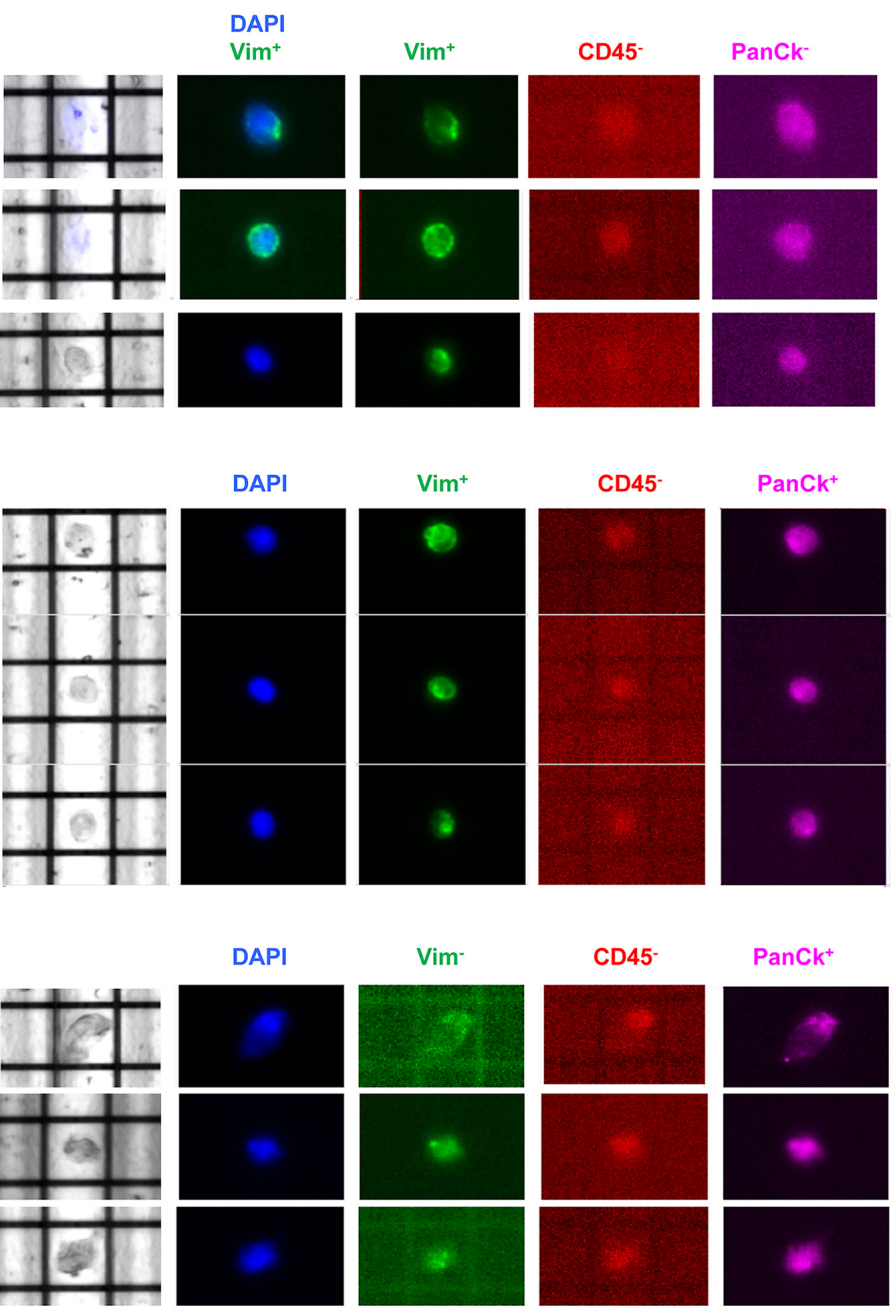
Representative CTCs isolated in soft-tissue sarcoma patients. CTCs were isolated by using a two-step process, including pre-enrichment by Parsortix^TM^, followed by visualisation and isolation with the DEPARarry^TM^ device after immunostaining. Three CTC populations were isolated: Vim^+^ CK^+^, Vim^-^ CK^+^, and Vim^+^ CK^-^. CTCs: Circulating tumour cells; Vim: vimentin; CK: cytokeratin; DAPI: 4',6-diamidino-2-phehylindole.

**Figure 2:**
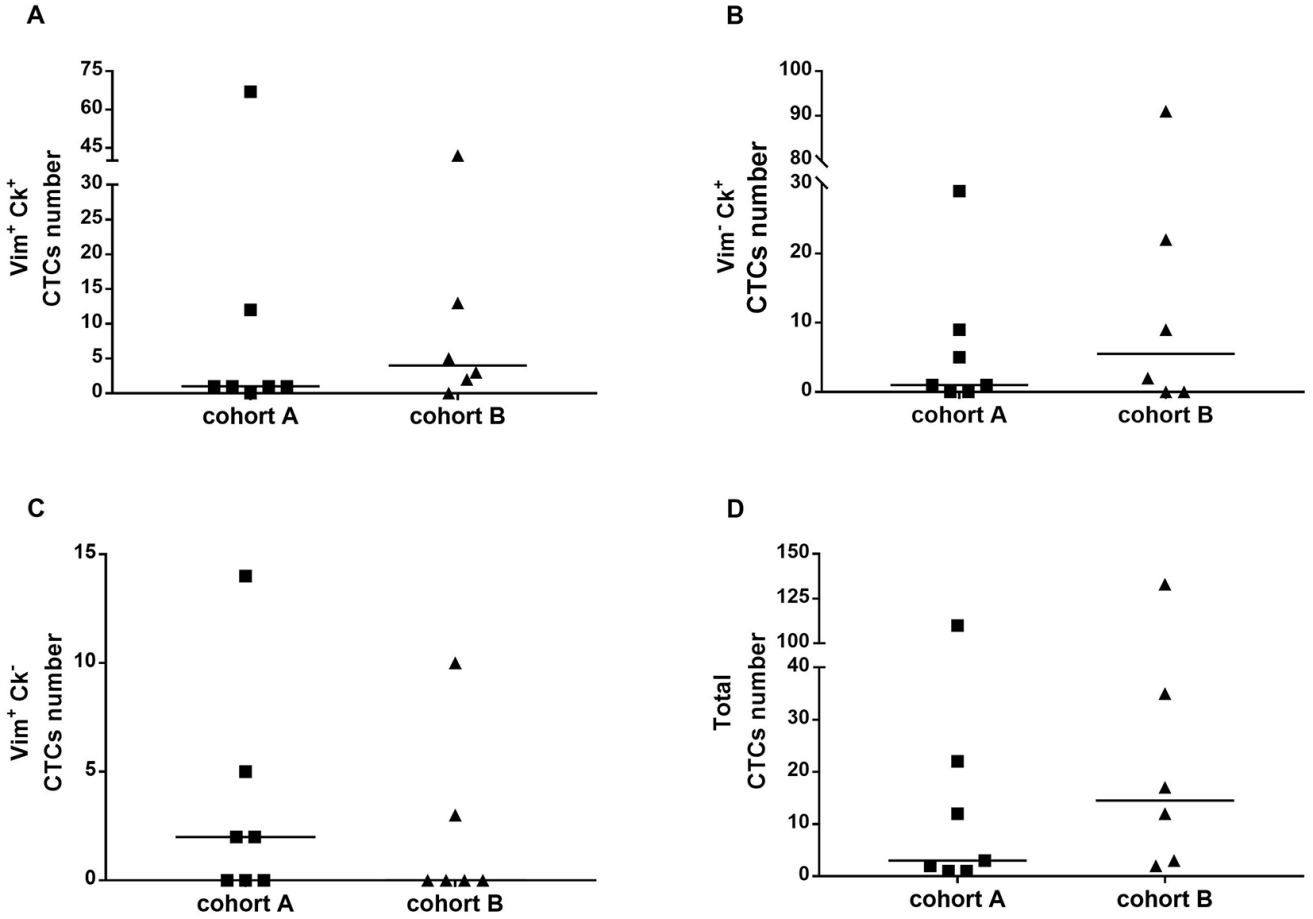
Plots comparing CTC populations between patients with localised (Cohort A) and advanced disease (Cohort B). CTCs from 20 mL of blood were pre-enriched using the Parsortix^TM^ microfluidic system and enumerated by DEParray^TM^ after immunostaining. CTCs were isolated from mesenchymal (Vim) and epithelial (Pan CK) biomarkers. (A) Vim^+^ CK^+^ CTCs; (B) Vim^-^ CK^+^ CTCs; (C) Vim^+^ CK^-^ CTCs; (D) Total CTCs. All CTCs were CD45^-^. CTCs: Circulating tumour cells; Vim: vimentin; CK: cytokeratin.

**Figure 3:**
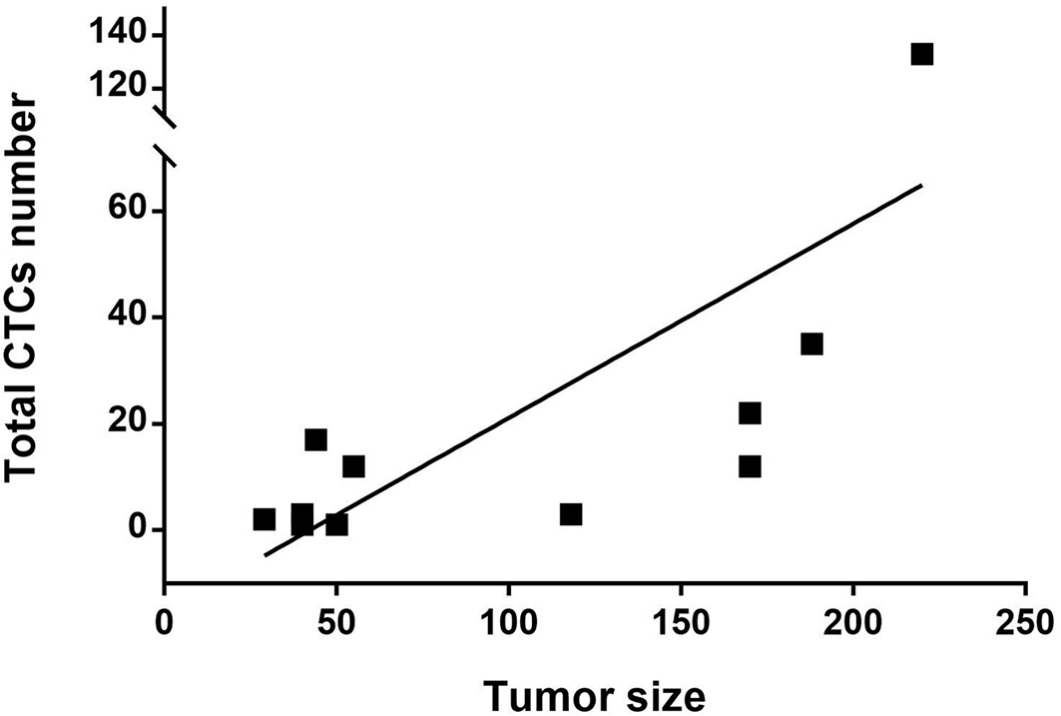
Plot showing the relationship between the total number of CTCs and the primary tumour size (mm). After pre-enrichment by Parsortix^TM^ device, Vim^+^ or/and pan CK^+^, CD45^-^ CTCs were isolated by DEParray^TM^. r_s_(8) = 0.786; *P* = 0.007. CTCs: Circulating tumour cells; Vim: vimentin; CK: cytokeratin.

Based on cell size and their expression of epithelial and mesenchymal markers, the total number of CTCs and 3 subpopulations were enumerated: Vim^+^ CK^+^, Vim^-^ CK^+^, and Vim^+^ CK^-^ CTCs [Figures 1 and 2]. CTCs were identified in all patients. There was a trend toward higher CTC numbers in patients with advanced disease, but this was not significant; the median number of total CTCs in patients with localised versus advanced disease was 3 (range 1-110) and 14.5 (range 2-133), respectively (*P* = 0.32). The number of CTCs was not associated with tumour grade [median number of total CTCs for Trojani grade ≤ 2 *vs.* 3 was 12 (range 1-133) and 10 (range 2-110), respectively (*P* = 0.77)]. However, there was a significant positive relationship between the total number of CTCs and the primary tumour size [r_s_(8) = 0.786; *P* = 0.007; Figure 3].

**Figure 4:**
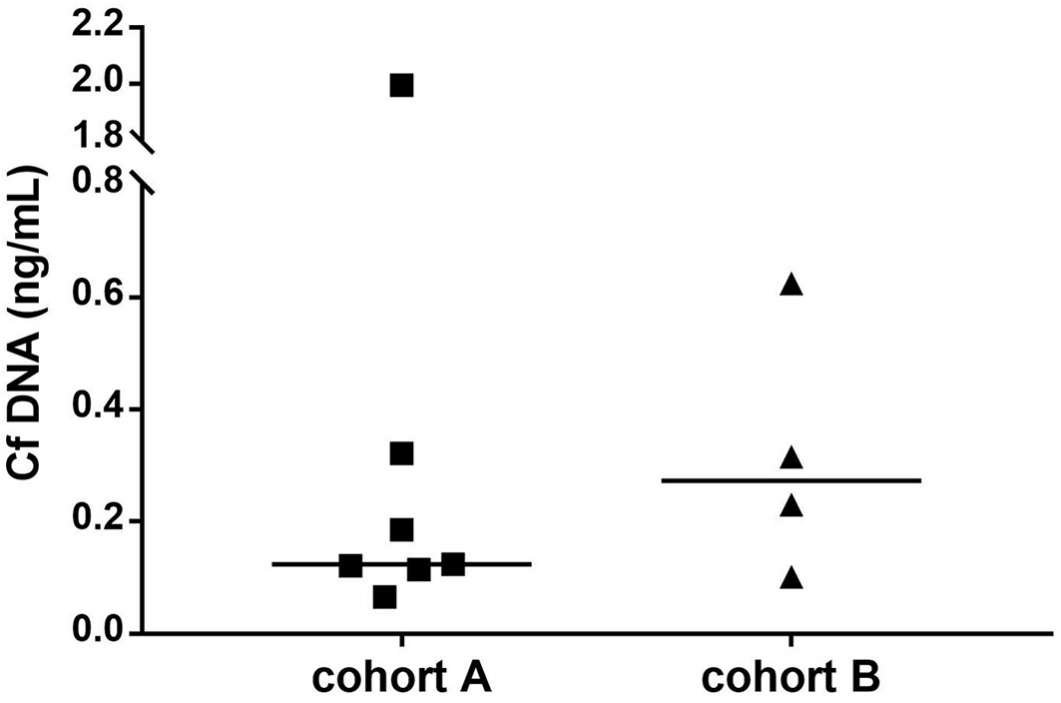
Plot comparing cfDNA (ng/μL) in localised (Cohort A) and advanced disease (Cohort B). cfDNA: Cell-free DNA.

cfDNA levels ranged from 0.07 ng/μL to 2.00 ng/μL with a median of 0.19 ng/μL. Levels were similar in the two patient cohorts [median cfDNA levels in localised disease was 0.12 ng/μL (range 0.07-2.00) and 0.28 ng/μL (range 0.10-0.62) in advanced disease; Figure 4]. No relationship was observed between either the cfDNA levels and the total number of CTCs [r_s_(11) = -0.20; *P* = 0.56], or the cfDNA levels and the primary tumour size [r_s_(7) = -0.25; *P* = 0.91].

The small sample size precluded any formal analysis, but there was no obvious relationship between either the total number of CTCs or cfDNA and disease-free survival.

## DISCUSSION

This pilot study adds to the growing literature on CTCs in STS. Consistent with other recent reports of CTCs in sarcoma, we found that CTCs were rare events with small numbers isolated per sample^[8-14]^.

As CTCs in sarcoma are rare, an efficient isolation process is needed for CTC capture. Our protocol included an initial enrichment step to concentrate the blood samples for CTCs utilising their physical properties. Parsortix^TM^ microfiltration cartridges were used to trap CTCs based on their increased size and lower deformability compared to normal cellular blood components (white and red blood cells). The enriched sample was then labelled using antibodies to target cell markers of interest. Initial studies of CTCs in sarcoma focused on translocation-associated sarcomas such as Ewing sarcoma^[15]^, with isolation of CTCs based on tumour cell expression of the characteristic fusion protein. Focusing on sarcomas with specific translocations is obviously restrictive, and thus, more ubiquitous sarcoma CTC markers have been sought. Vim, a cytoskeleton component that is universally expressed in mesenchymal cells, has since been proposed as a pan-sarcoma CTC marker^[6]^. However, sarcoma cells may also express cytokeratins, particularly within a subpopulation of tumour cells undergoing mesenchymal-to-epithelial transition as part of the metastatic process^[16]^. Thus, an isolation process identifying sarcoma CTCs exclusively by Vim expression may omit other CTC subpopulations of interest. We explored the expression of both Vim and CK markers, using the DEPArray^TM^ to isolate and quantify these cell populations from the enriched blood samples. One of the strengths of our study was to capture data on a broad population of CTCs, and a further advantage of our process is that the DEPArray^TM^ enables the separation of different cell populations (e.g., Vim^+^/CK^-^ *vs.* Vim^+^/CK^+^) for further downstream analysis and characterisation. We observed an important heterogeneity of CTCs with various expression of biomarkers that may reflect a snapshot of the composition of the localised or metastatic tumour at a given time^[5]^. Unfortunately, limited resources prohibited further molecular analyses in the current study.

We collected CTCs at first presentation before initiation of any treatment - surgery, radiotherapy, or systemic treatment. Whether CTC numbers or other characteristics change in response to treatment is unclear. In keeping with some studies, our data support CTC numbers correlate with tumour burden^[8-10]^. However, other studies have found no such relationship, nor any association with treatment response^[11,13]^. To date, published studies on CTCs in sarcoma have involved relatively small patient cohorts, and different CTC isolation techniques have been employed, making robust cross-study comparisons difficult. The clinical value of CTCs in sarcoma thus remains uncertain and larger studies are needed to investigate this further.

A key clinical challenge in sarcoma is identifying patients with high-risk localised disease who may benefit most from intensive adjuvant chemotherapy. Existing prognostic calculators already in clinical use, such as the Sarculator^[17]^, currently rely on clinical characteristics such as tumour size, grade, and histological subtype. Potentially, CTCs could be useful as a prognostic marker to further refine patient selection for adjuvant chemotherapy, but clearly, further and larger studies are needed to explore this possibility. One of the largest reported studies to date of CTCs in sarcoma was a translational endpoint within the TAPPAS study, a randomised phase III study of TRC105 +/- pazopanib for advanced angiosarcoma^[18]^. While CTC numbers did not change in response to treatment, it is noteworthy that CTCs were identified in the majority of the evaluated patients^[19]^. Our study included one patient with localised angiosarcoma, who had a high baseline count of CTCs and subsequently quickly relapsed. Angiosarcoma is a very aggressive sarcoma and perhaps represents a particularly promising subtype for further CTC research. In particular, serial blood samples collected before and during treatment may serve as a liquid biopsy, enabling real-time analysis of tumour cell response to treatment and the development of drug resistance.

A potential limitation of our workflow is the loss of smaller CTCs during the enrichment step, although previous research indicates a high recovery rate^[7]^. The main limitation, however, is the small number of patients included in this pilot study, which prevents us from drawing conclusions about the clinical significance of CTCs in sarcoma. Nonetheless, our pilot study demonstrated the feasibility of our process for isolating CTCs from patients with STS. Our methodology enables the capture and separation of CTCs into subpopulations for additional downstream analysis, and thus provides a platform for larger studies to explore both the clinical utility of CTCs in sarcoma and molecular investigations into the metastatic process in sarcoma.

## DECLARATIONS

### Availability of data and materials

All data generated or analysed in this study are included in this manuscript.

### Copyright

© The Author(s) 2024.
